# Structural alterations of branched *versus* linear mixed-surfactant micellar systems with the addition of a complex perfume mixture and dipropylene glycol as cosolvent†

**DOI:** 10.1039/d2ra00688j

**Published:** 2022-05-17

**Authors:** Marzieh Mirzamani, Marc Flickinger, Arnab Dawn, Vinod Aswal, Boualem Hammouda, Ronald L. Jones, Edward D. Smith, Harshita Kumari

**Affiliations:** James L. Winkle College of Pharmacy, University of Cincinnati Cincinnati OH 45219-0004 USA kumariha@ucmail.uc.edu; The Procter & Gamble Company 8700 Mason Montgomery Road Mason OH 45040 USA; Bhabha Atomic Research Center Mumbai Maharashtra India; NIST Center for Neutron Research, National Institute of Standards and Technology 100 Bureau Drive Gaithersburg MD 20899-6102 USA

## Abstract

Personal care products commonly contain perfume mixtures, consisting of numerous perfume raw materials (PRMs), and cosolvents. The lipophilicity and structure of an individual PRM is known to affect its localization within the surfactant self-assembly as well as the micellar geometry. However, because multiple PRMs are used in formulations, significant intermolecular interactions between the PRMs and between the PRMs and the surfactant tail may also influence the location of the PRMs and their effects on the self-assembly. Herein, two anionic/zwitterionic mixed-surfactant systems (sodium trideceth-2 sulfate (ST2S)/cocamidopropyl betaine (CAPB) and sodium laureth-3 sulfate/CAPB) were formulated with a cosolvent (dipropylene glycol (DPG)) and 12 PRMs of varying structures and lipophilicities. This 12 PRM accord is simpler than a fully formulated perfume but more complex than a single perfume molecule. The geometric variations in the self-assemblies were evaluated using small-angle neutron scattering, perfume head space concentrations were determined using gas chromatography-mass spectrometry, and perfume localization was identified using NMR spectroscopy. The addition of the perfume accord caused enlargement of the micelles in both surfactant systems, with a greater change observed for ST2S/CAPB formulations. Furthermore, the addition of DPG to ST2S/CAPB resulted in micelle shrinkage. The micelle geometries and PRM localization in the micelles were affected by the degree of branching in the surfactant tail.

## Abstract 2

Personal care products commonly contain perfume mixtures, consisting of numerous perfume raw materials (PRMs), and cosolvents. Depending on the molecular structures of the additives and surfactants, the geometry of the colloidal structures can be affected.

## Graphical abstract

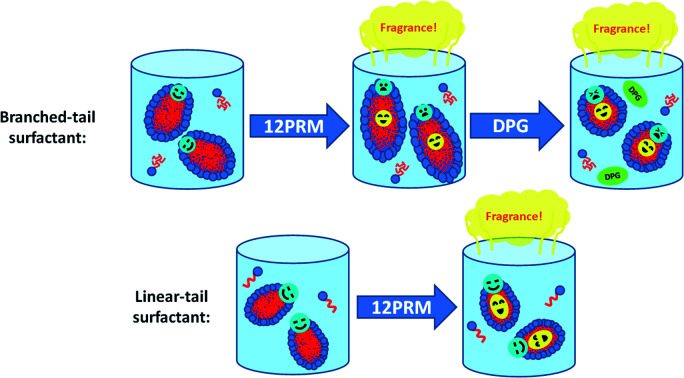

## Introduction

Odiferous molecules are ubiquitous in everyday life; they are used in foods to enhance flavor; in house-cleaning products, cosmetics and perfumery, and personal care products to impart pleasant aromas; in cleansers when added as an essential oil; and in aromatherapy for beneficial psychological effects.^1–4^ Because fragrance is a primary factor in consumer purchasing choice^5,6^ and requires the use of expensive ingredients, efficient ingredient deposition onto surfaces is crucial to minimize product loss during rinse-off.

Perfumes are commonly classified by their functional groups, such as aldehydes, alcohols, and phenols, and by their log *P* values, which denote the lipophilicity or hydrophilicity of a perfume raw material (PRM).^1,2^ Their inherent high volatility, hydrophobicity, and susceptibility to oxidation upon storage render formulation rather difficult. Methods such as spray drying, extrusion, coacervation, and emulsification are used to appropriately encapsulate and release perfumes from colloidal domains. Gas chromatography-mass spectrometry (GC-MS) is traditionally used to assess the correlation between the log *P* values of perfumes and their release profile. However, ambiguity about the colloidal domains of formulations complicates such analyses.

This complexity is compounded by the use of numerous PRMs to generate a fragrance within a formulation. As the fragrance is generated based on a complex PRM mixture, the relationship of the fragrance with the overall formulation is not straightforward.^3^ Our previous work with a 3 PRM accord in a mixed-surfactant system with a cosolvent^7^ revealed that surfactant, cosolvent, and oil combinations were not conducive to the formation of single-phase microemulsions. NMR analysis showed that the 3 PRMs were preferentially localized within the micelle core, regardless of their lipophilicities, indicating that perfume–perfume interactions strongly influence the location of a PRM within a surfactant self-assembly. Additionally, small-angle neutron scattering (SANS) measurements showed that increasing the concentration of the 3 PRM accord caused continual growth of the self-assemblies, which maintained spherical or ellipsoidal geometries at high surfactant concentrations. This behavior contrasts with the planar, vesicular, or worm-like structures that are expected to form based on the lipophilicities and molecular structures of specific PRMs in the system. Hence, in the present work, we created a more complex perfume accord consisting of 12 PRMs with various structures and physical properties, which approaches the complexity of a full perfume formulation and has a good presence in the headspace. It is envisioned that this approach will advance the understanding of how an actual perfume mixture influences the aggregation properties of rinse-off systems at concentrations that better reflect those of personal care products, and whether the PRMs are again preferentially localized somewhere within the surfactant self-assembly.

Specifically, we studied the location of the 12 PRMs and the colloidal domains in two mixed-surfactant systems: sodium trideceth-2 sulfate (ST2S)/cocamidopropyl betaine (CAPB) and sodium laureth-3 sulfate (SLE3S)/CAPB. These surfactant systems were chosen as representative of the mixed-surfactant systems commonly used in personal care products for mildness. Moreover, these systems allowed us to expand upon our previous work^7^ by investigating the influence of surfactant structure (branched (ST2S) *versus* linear (SLE3S)) on perfume localization. Furthermore, we determined the concentration of each PRM in the headspace of each mixed-surfactant system. In addition, we investigated the effects of perfume concentration and the addition of a cosolvent (dipropylene glycol; DPG) on the mixed-surfactant systems. The addition of a cosolvent is of particular interest, as it reduces the cohesive energy density and dielectric constant of the medium by affecting polar intermolecular forces including hydrogen bonding.^8,9^ DPG is commonly used as a cosolvent in detergents, personal care products, and cosmetics owing to its low toxicity and high dielectric constant.^8^ It is also commonly used in perfumery as a solvent and diluent because of its high boiling point, transparency, and lack of odor. Although there are reports pertaining to the effect of cosolvent,^8,10–13^ perfume accords,^2,14^ and individual perfumes, in surfactant systems,^15–17^ information on the combined effects of a cosolvent and a perfume accord on a surfactant system is limited,^14^ likely because of the complexity associated with the use of multiple additives.

We used SANS to elucidate the size and shape of the self-assemblies in solution.^18–22^ In addition, we used NMR spectroscopy to investigate the location of the PRMs and GC-MS to study perfume release from the formulations. The use of a moderately complex formulation and perfume accord allowed for a more realistic investigation of how a fragrance composition consisting of multiple PRMs affects the structure of the self-assemblies.

## Methods

### Sample preparation

**Scheme 1:**
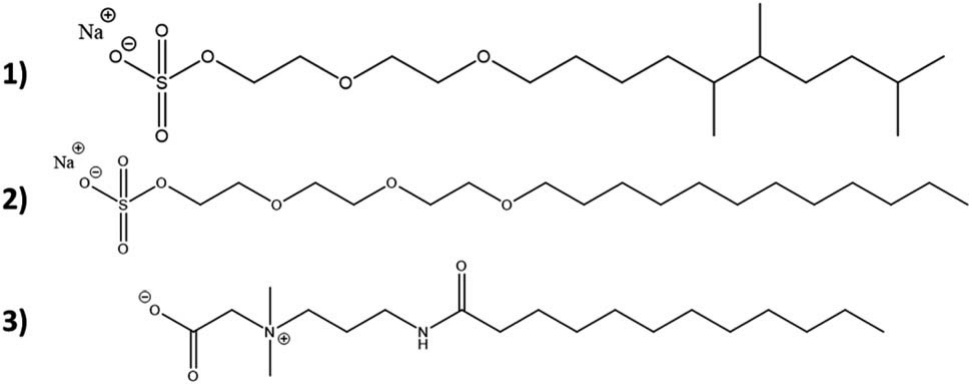
Surfactant structures: (1) sodium trideceth-2 sulfate (ST2S), (2) sodium laureth-3 sulfate (SLE3S), and (3) cocamidopropyl betaine (CAPB). The tested samples contained either a mixture of ST2S and CAPB (ST2S/CAPB, branched-tail surfactant system) or a mixture of SLE3S and CAPB (SLE3S/CAPB, linear-tail surfactant system).

All raw materials were provided by Procter & Gamble. In total, eight samples were tested. The compositions of the SANS and NMR samples are detailed in Table 1. The GC-MS samples had the same compositions, except that only H_2_O was used instead of H_2_O/D_2_O mixtures. The first five samples contained ST2S, a branched-tail surfactant, and the remaining three samples contained SLE3S, a linear-tail surfactant. In addition to these primary surfactants, each system also included CAPB. The surfactant structures are shown in Scheme 1. Samples 1–3 and 6–8 had increasing amounts of the perfume accord, whereas samples 2, 4, and 5 had increasing amounts of DPG and a constant 0.5 wt% perfume accord. The perfume accord used in this study consisted of 12 PRMs, chosen to cover a wide range of log *P* values and molecular structures (Table 2).

**Table 1:** Compositions of SANS and NMR samples (for GC-MS samples, D_2_O was replaced with H_2_O)

Component (wt%)								
Material	Sample 1	Sample 2	Sample 3	Sample 4	Sample 5	Sample 6	Sample 7	Sample 8
Sodium trideceth-2 sulfate (ST2S)	6.402	6.402	6.402	6.402	6.402	—	—	—
Sodium laureth-3 sulfate (SLE3S)	—	—	—	—	—	6.750	6.750	6.750
Cocamidopropyl betaine (CAPB)	1.098	1.098	1.098	1.098	1.098	0.750	0.750	0.750
Citric acid	0.110	0.110	0.110	0.110	0.110	0.110	0.110	0.110
Dipropylene glycol (DPG)	0.000	0.000	0.000	1.500	3.000	0.000	0.000	0.000
Perfume	0.000	0.500	1.000	0.500	0.500	0.000	0.500	1.000
H_2_O	7.009	6.509	6.009	7.509	6.009	27.670	27.170	26.670
D_2_O	85.381	85.381	85.381	82.881	82.881	64.720	64.720	64.720

**Table 2:** Composition of perfume accord and selected physical properties of the components

Material	Structure	Content (wt%)	Molecular weight (g mol^−1^)	*c* log *P*
Benzyl acetate	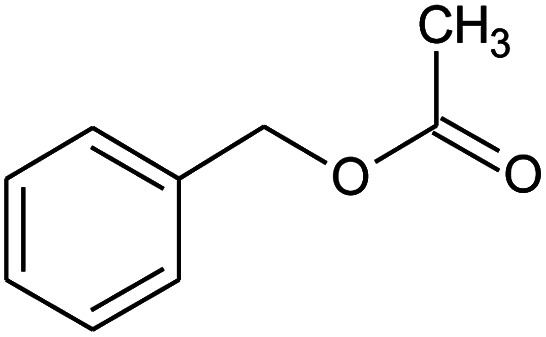	5	150.18	1.7
Dihydromyrcenol	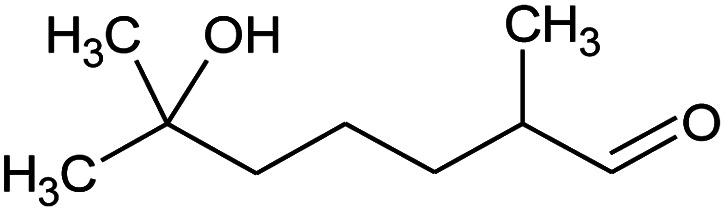	10	156.3	3.08
Phenylethyl alcohol	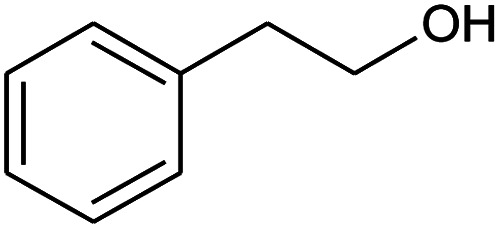	5	122.17	1.32
Florosa Q (pyranol)	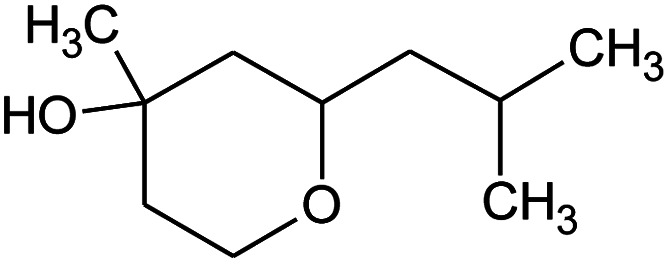	5	172.3	2.46
β-Ionone	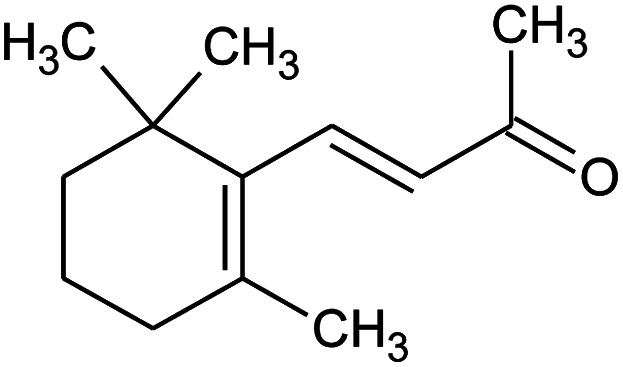	10	192.3	4.02
Undecavertol	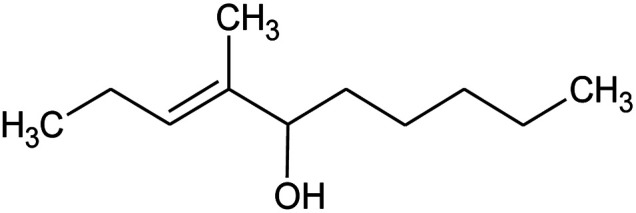	10	170.3	3.06
Ambrox (Ambronat)	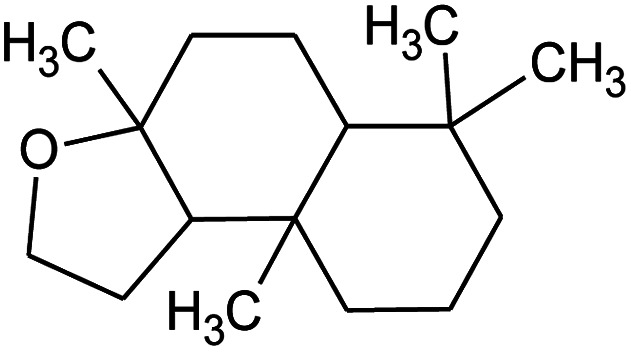	5	236.4	4.58
Heliotropin	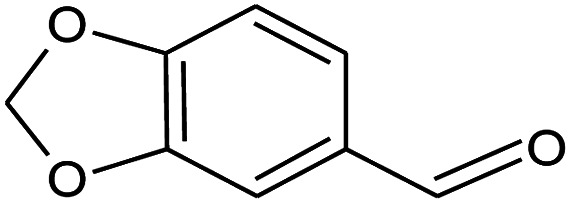	5	150.1	1.43
γ-Decalactone	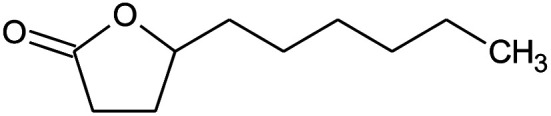	10	170.2	3.23
Methyl dihydrojasmonate	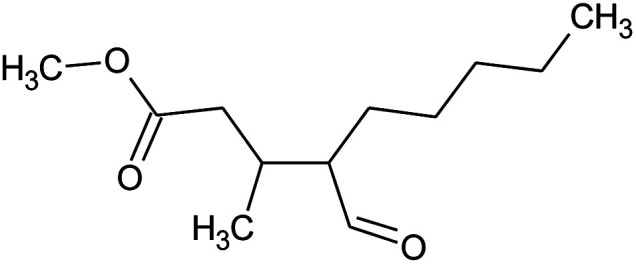	15	226.3	3.01
Hexyl cinnamic aldehyde	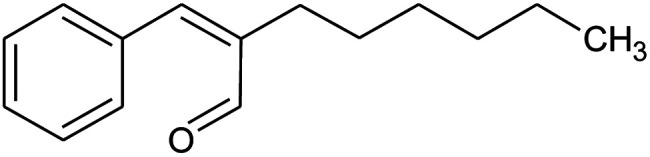	10	216.3	4.3
Galaxolide (hexamethylindanopyran)	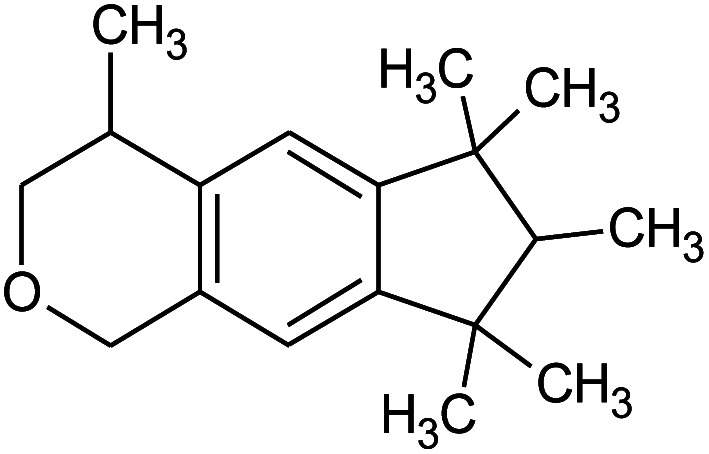	10	258.4	5.43

### SANS studies

SANS studies were conducted at the NIST Center for Neutron Research (NCNR) in Gaithersburg, MD. In the SANS samples (Table 1), D_2_O was used to maximize the contrast between the solvent and the micelles. However, because the surfactants all included known amounts of H_2_O, the solvent phase was mostly D_2_O mixed with some H_2_O. Samples 1–3 were run on the 30 m NG7-SANS beam line, whereas samples 4–8 were run on the 10 m NGB-SANS beam line, which is operated as part of the nSoft Consortium. The samples were placed in titanium cell holders with quartz windows, which were separated by a 1 mm sample gap. Different neutron wavelength (*λ*) and sample-to-detector distance (SDD) configurations were used to achieve the desired *q*-range, where the scattering vector *q* = (4π/*λ*)sin(*θ*/2)  and *θ* is the scattering angle. Samples 1–3 were irradiated with neutrons of wavelength *λ* = 6 Å at SDDs of 1 m (high-*q* configuration) and 4 m (mid-*q* configuration), and with neutrons of wavelength *λ* = 8.09 Å at an SDD of 15.3 m (low-*q* configuration) to cover a wide *q*-range of 0.0027–0.5568 Å^−1^. The data collection times were 5 min, 20 min, and 1 h for the high-, mid-, and low-*q* ranges, respectively. For samples 4–8, the high-*q* configuration was *λ* = 5 Å with an SDD of 1.2 m, the mid-*q* configuration was *λ* = 5 Å with an SDD of 4.6 m, and the low-*q* configuration was *λ* = 12 Å and an SDD of 4.6 m, which covered a *q*-range of 0.00318–0.5666 Å^−1^. The data collection times were 10, 20, and 45 min for the high-, mid-, and low-*q* ranges, respectively. All sample data were collected at 25 °C.

Using the data reduction macros developed for Igor Pro by the NCNR,^23^ the data were reduced by correcting for background scattering, detector resolution and sensitivity, and beam transmission. Then, the data were radially averaged to obtain the absolute scattering intensity, *I*(*q*). The high-, mid-, and low-*q* data were combined to obtain the complete data set. Each data set was analyzed in SasView 5.0 ref. (24) using the smeared-resolution ellipsoid form factor coupled with the Hayter–Penfold rescaled mean spherical approximation (MSA) structure factor model to account for electrostatic effects. The equations used for the ellipsoid form factor in SasView are given by Feigin and Svergun.^25^ The equations describing the structure factor were developed by Hayter and Penfold^26^ and by Hansen and Hayter.^27^ A polydispersity term was included in the model for the equatorial radius (*R*_e_, perpendicular to the axis of rotation) using a Schulz distribution. Finally, the decoupling approximation, which was developed by Hayter and Penfold^28^ and discussed further by Kotlarchyk and Chen^29^ to correct for errors in the structure factor calculation caused by polydispersity and nonsphericity, was also used in modeling the data.

The magnitudes of the polar radius (*R*_p_, parallel to the axis of rotation) and *R*_e_ in the ellipsoid model determine whether the ellipsoid is oblate or prolate. For *R*_e_ > *R*_p_, the ellipsoid is oblate (disc-like); for *R*_p_ > *R*_e_, the ellipsoid is prolate (melon-shaped); and for *R*_p_ = *R*_e_, the ellipsoid is spherical. The data were modeled using both the oblate and prolate ellipsoid cases, and which type of ellipsoid best described the data was determined based on the minimized residuals and the visual fit quality. Particular attention was paid to minimizing the residuals in the high-*q* region, as this region is most sensitive to the form factor and least affected by the structure factor. If both cases fit the data similarly, then the model with the smallest sqrt(*χ*^2^/*N*) was chosen as the best fit. The solvent scattering length density (SLD) was calculated and fixed based on the weighted average of D_2_O, H_2_O, citric acid, and DPG in each sample. The micelle (*i.e.*, particle) SLDs were also calculated and fixed to the weighted average of the primary surfactant (*i.e.*, ST2S or SLE3S), CAPB, and the perfume accord, if applicable. To simplify the SLD calculations, an average molecular formula and density were calculated for the perfume accord based on the weighted average of its components. The SLDs for the solvents and micelles as well as the parameters used to calculate them can be found in the ESI.†

### GC-MS studies

GC-MS samples were prepared at room temperature two days before analysis to allow for equilibration. The components were added in the following order: ST2S or SLE3S, CAPB with 3 wt% citric acid, DPG, water, and the PRM accord. The sample was then vortexed until the components were thoroughly blended and set aside until testing. The GC-MS data were obtained using an Agilent Model 6890 gas chromatograph fitted with a Gerstel MPS-2 autosampler, a 0.75 mm ID SPME injection port liner (Supelco, Bellefonte, PA, USA), and a J&W DB5-MS GC column with an ID of 30 m × 0.25 mm and a film thickness of 1.0 μm (Agilent Technologies, Inc., Wilmington, DE, USA). The detector was a Model 5973 mass selective detector (Agilent Technologies, Inc., Wilmington, DE, USA) with a source temperature of approximately 230 °C and a MS Quad temperature of approximately 150 °C. Ultra-pure helium was used as the carrier gas with a flow rate of 1 mL min^−1^.

Each sample (3 g) was placed in a clean 20 mL headspace vial with a magnetic stir bar. After the vials were closed with PTFE septum caps, the samples were stirred and allowed to equilibrate at room temperature for at least 30 min. The sample vials were then placed in an autosampler tray. Each sample vial was automatically placed in the sampling chamber, where it equilibrated at 30 °C for 1 min. The sampling time was 1 min., during which the sample was stirred at 500 rpm and taken up by a 50/30 μm, 24 ga, 1 cm long DVB/CAR/PDMS SPME fiber assembly. The sample was injected at 270 °C, and the GC-MS analysis was begun after allowing the sample to desorb from the SPME assembly for 5 min. The temperature was initially held at 50 °C for 30 s, and then increased to 275 °C at a rate of 8 °C min^−1^ and held for 2.5 min. The individual PRMs were identified using MS spectral libraries from John Wiley & Sons and the National Institute of Standards and Technology, which were purchased and licensed through Agilent. The chromatographic peaks of specific ions were integrated using the MassHunter software (Agilent Technologies, Inc., Wilmington, DE, USA).

### NMR studies

^1^H-NMR spectroscopy was performed using a Bruker AV 400 MHz spectrometer (Billerica, MA, USA). The NMR samples were prepared 3 days before analysis using D_2_O as the solvent. The H_2_O peak at 4.69 ppm was used as a reference for data analysis.

## Results and discussion

### Effect of 12 PRM accord addition

**Fig. 1:**
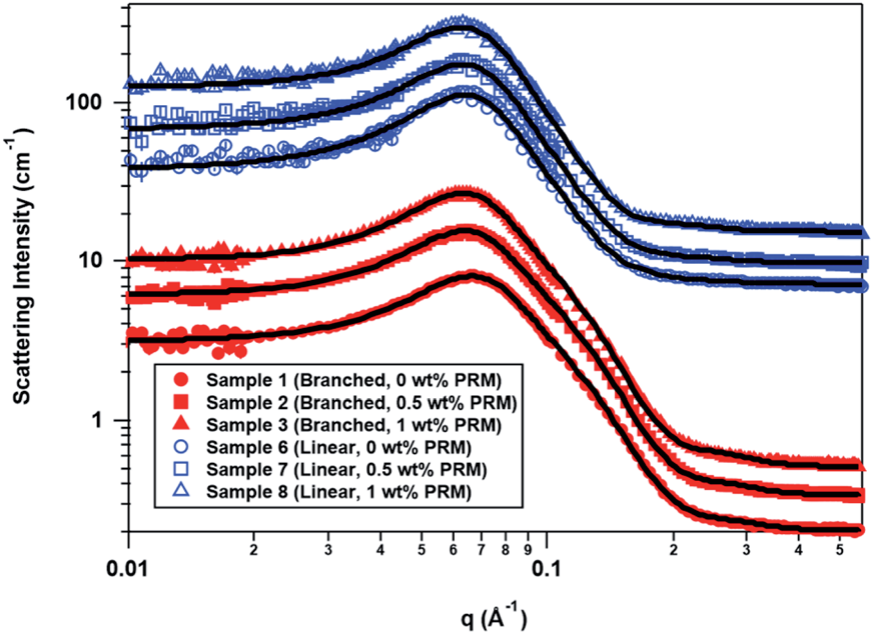
SANS data overlays for branched-tail (red, solid symbols) and linear-tail (blue, empty symbols) mixed surfactant systems as a function of perfume concentration. The data sets are offset to improve clarity, with the 0.5 and 1 wt% perfume curves offset from the 0 wt% perfume curve by powers of 1.5, and the blue curves offset from the red curves by an additional factor of 20. The solid black lines are the model fits to the data.

Mixed micelles composed of a primary surfactant (ST2S or SLE3S) and a secondary surfactant (CAPB) will form because the total surfactant concentration of 7.5 wt% is above the critical micelle concentration. The hydrophobic PRM accord is expected to localize within the micelles, while DPG is expected to primarily locate in the aqueous phase based on literature.^8^ The SANS data modeling results are summarized in Table 3 and the individual fitting parameters for each sample are available in the ESI.† The SANS fitting results in Table 3 show how the micelle radii and volumes of the ST2S and SLE3S systems vary with the perfume content, and Fig. 1 shows an overlay of the SANS data and the model fits as functions of perfume concentration and mixed-surfactant system.

**Table 3:** Volume fractions (calculated from the amounts of surfactant and perfume in the system, and as determined by SANS), dimensions, and volume of branched-tail and linear-tail mixed surfactant self-assemblies as a function of perfume concentration

Surfactant tail structure	Perfume concentration (wt%)	Calculated vol. fraction	Vol. fraction (SANS)	*R*_p_ (Å)	*R*_e_ (Å)	Volume (Å^3^)
Branched	0	0.07	0.08	54.82	18.21	76 140.5
	0.5	0.07	0.10	66.86	18.70	97 936.5
	1	0.08	0.11	68.20	19.43	107 856
Linear	0	0.07	0.08	41.08	22.91	90 316.9
	0.5	0.07	0.08	42.95	23.23	97 084.6
	1	0.08	0.09	44.68	23.99	107 711.5

### Effect of DPG addition

The self-assembly volume fractions determined by SANS were generally similar to the volume fractions calculated from the amount of each component expected to be in the dispersed phase (ST2S or SLE3S, CAPB, and perfume). However, once perfume was added to the ST2S/CAPB system, the volume fraction from SANS was somewhat higher than expected, as if the perfume added disproportionately more volume to the dispersed phase. This behavior suggests that the perfume formed a small droplet surrounded by surfactant monomers in the core of the ST2S/CAPB micelle instead of being solubilized by the surfactant tails, as presumed to occur in the SLE3S/CAPB system. As *R*_p_ > *R*_e_ for the branched-tail and linear-tail mixed-surfactant systems, prolate ellipsoids were formed for both systems. Interestingly, the size and geometry of the ST2S/CAPB micelles were more strongly affected by perfume addition than those of the SLE3S/CAPB micelles. Specifically, *R*_p_ increased for both systems as perfume was added, sharply increasing in the ST2S/CAPB system but gradually increasing in the SLE3S/CAPB system, whereas *R*_e_ remained virtually constant in both systems. As a result, the volume change of the ST2S/CAPB micelles upon perfume addition was larger than that of the SLE3S/CAPB micelles.

As perfume accords are water-insoluble, surfactant micelles are required for solubilization in aqueous environments. The ST2S surfactant forms smaller micelles because the branched-tail structure causes the alkyl chains to pack differently in the core than the linear chains of SLE3S.^30^ This packing is reflected in the *R*_p_ values, which show that the tails pack together to form elongated micelles in the ST2S/CAPB system. The tails may not be packed quite as tightly along the long axis of the micelle, which could allow the micelle to elongate further as perfume is taken up. Because of its longer linear-tail and extended head group, the SLE3S monomer is longer than the ST2S monomer, which explains why the SLE3S/CAPB micelles expand more gradually with perfume addition than the ST2S/CAPB micelles. When the 3 PRM accord and the present 12 PRM accord were added to a concentrated ST2S/CAPB system, self-assembly began with the formation of spherical structures that enlarged and eventually formed oblate ellipsoids in both cases.^7,31^ Although the oil composition influences the extent to which a microemulsion can be diluted and the range of compositions which form microemulsions,^31^ the differences in size and geometry between the present work and our past work could be caused by a few key differences—namely, the total surfactant concentration (7.5 wt% *versus* 20–32.5 wt%, respectively), and the ratio of perfume : surfactant : DPG, or both.

**Fig. 2:**
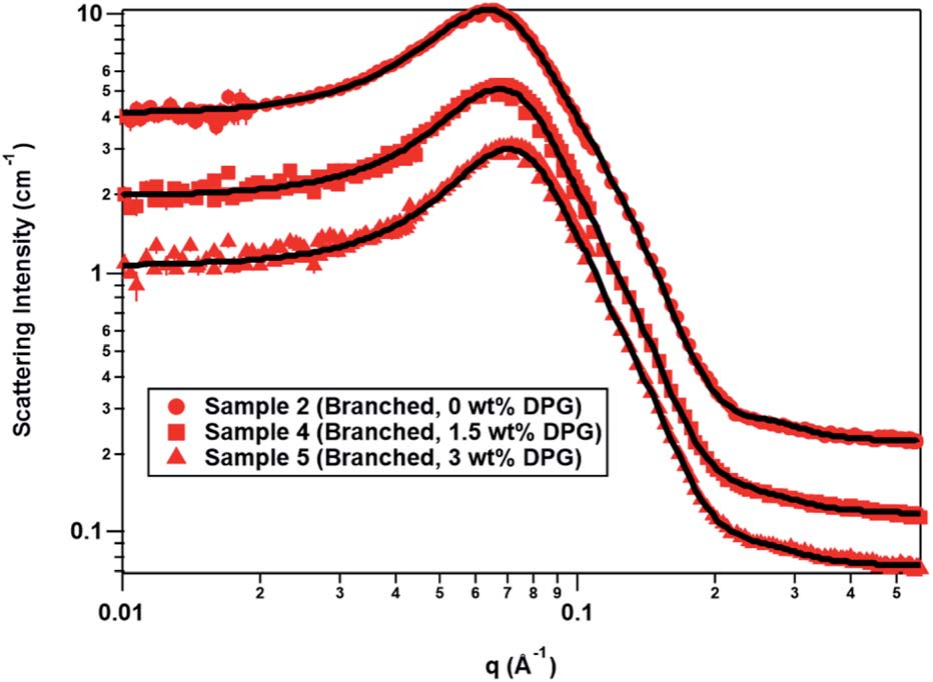
SANS data overlays for the branched-tail mixed surfactant system as a function of DPG concentration. The data sets are offset to improve clarity, with the 1.5 and 3 wt% DPG curves offset from the 0 wt% DPG curve by powers of 2/3. The solid black lines are the model fits to the data. Location of perfume accord in micelles.

Table 4 shows the change in the ST2S/CAPB volume fraction, micelle radii and volume with the addition of DPG, and Fig. 2 shows the overlay of the scattering data and model fits. The volume fractions calculated from the amounts of ST2S, CAPB, and perfume in the system were constant because the surfactant and perfume concentrations did not change; however, the volume fractions determined from SANS decreased from 0.10 to 0.07 as DPG was added. With respect to the micelle radii, although the micelles were prolate ellipsoids at all DPG concentrations (*R*_p_ > *R*_e_), the micelles became less elongated with the addition of DPG owing to a decrease in *R*_p_. Specifically, the decrease in micelle volume as a function of DPG concentration was due to shrinkage along one axis (*R*_p_), resulting in less elongated micelles. Notably, this trend is contrary to that obtained with perfume addition. The change in *R*_e_ with DPG addition was small, as also observed upon perfume addition. DPG aids in solubilizing the perfume oil in the continuous phase, which explains the decreases in the volume fraction, micelle radii, and micelle volume, as less perfume needs to be solubilized inside the micelles. Recent work on the effects of DPG on the self-assembly of a cationic cetyltrimethylammonium tosylate (CTAT) system^11^ and a sodium laureth-1 sulfate (SLE1S)/CAPB system^8^ revealed that DPG reduces the micelle size. With DPG primarily located in the aqueous phase, the solvent dielectric constant is decreased, which increases electrostatic repulsions between the head groups, resulting in shrinkage of the micelle radius through an increase in the curvature of the micelle surface.^8^ Apparently, the known effects of DPG still apply to the overall perfume accord, despite its complexity.

**Table 4:** Volume fractions (calculated from the amounts of surfactant and perfume in the system, and as determined by SANS), dimensions, and volume of the branched-tail mixed surfactant system as a function of DPG concentration

DPG concentration (wt%)	Calculated vol. fraction	Vol. fraction (SANS)	*R*_p_ (Å)	*R*_e_ (Å)	Volume (Å^3^)
0	0.07	0.10	66.86	18.70	97 936.5
1.5	0.07	0.08	45.74	19.25	71 002.7
3	0.07	0.07	40.88	18.89	61 082.7

As noted above, the geometry and sizes of the colloidal domain in this work differ from those in our previous work.^31^ The 5 branched-tail mixed micelle systems studied in this work can be approximately located in the surfactant corner of the tertiary phase diagram at 90 wt% water (Fig. S1† in the ESI†) that was developed in our previous work,^31^ whereas the samples made as part of that work are located along the 1 : 1 surfactant : DPG line. This means that the 5 present samples contain more surfactant than the previous samples^31^ (7.5 wt% *vs.* 3.7–5 wt%), which is why there are also higher volume fractions of micelles in the present systems. Additionally, the amount of DPG in the current samples is overall lower than it was in the previous samples^31^ (0–3 wt% *vs.* 3.7–5 wt%), which further results in a surfactant : DPG ratio that is heavily skewed toward surfactant in this work. Here, the micelles not only shrank with DPG concentration, but also the aspect ratio decreased so that the micelles became less prolate; in comparison, the structures observed in our previous work^31^ were oblate ellipsoids. This could suggest that the surfactant : DPG ratio, in addition to the overall DPG concentration, influences the micelle geometry as well as its size.

To obtain insights into perfume localization, we performed NMR studies on the mixed-surfactant systems in presence and absence of the PRM accord. The NMR peak shifts for the two surfactant systems with varying perfume concentrations were compared (Table 5 and Fig. S1†). The changes associated with each signal were small because of the very low perfume content. Nevertheless, these changes provided useful information about the preferential distribution of perfumes in the micelles.^7^ In the SLE3S/CAPB system, protons ‘1’, ‘2’, and ‘3’ exhibited shielding effects upon perfume addition, indicating the localization of the perfume molecules near these surfactant protons. Similar effects were observed in the ST2S/CAPB system, but the shielding effect was more prominent for hydrophobic protons ‘1’ and ‘2’ than the more hydrophilic proton ‘3’. This observation implies that the PRMs (irrespective of their individual log *P* values) are preferentially located near the micelle core. This finding is very similar to our recent results for a 3 PRM system,^7^ but the implication here is more significant, as the increased complexity in the system with 12 PRMs did not alter the perfume localization tendency. Furthermore, this finding differs from the behavior reported for individual PRMs in earlier studies.^17,32,33^ Apparently, the methyl group branches in the ST2S tail facilitate the retention of perfume molecules near the micelle core by creating an efficient hydrophobic pocket. Although Fieber *et al.* showed that the surfactant composition and molecular structure of cosurfactants influences the partitioning of perfume molecules,^34^ the collective findings from our previous 3 PRM system and the present 12 PRM system clarify the importance of the tail nature and conformation of the primary surfactant in driving the localization of PRM accords within micelles. In addition, weak (because of low PRM concentrations) intermolecular interactions among different PRMs become operative in mixtures. It is worth noting that the log *P* values of individual PRMs become less relevant in dictating the localization of PRMs in the micelle.

**Table 5:** NMR peak shifts of primary surfactants (ST2S, and SLE3S) as a function of increasing perfume concentration (a negative sign indicates an upfield shift). The molecular structure and signal assignments for SLE3S are shown in (a), and those for ST2S are shown in (b)

	Perfume concentration (wt%)	Peak shift (*Δ* ppm)			
		1	2	3	4
SLE3S/CAPB	0	0	0	0	0
	0.50	−0.01	−0.01	−0.01	0
	1	−0.02	−0.02	−0.02	0
ST2S/CAPB	0	0	0	0	0
	0.50	−0.01	−0.01	−0.01	0
	1	−0.02	−0.02	−0.01	0
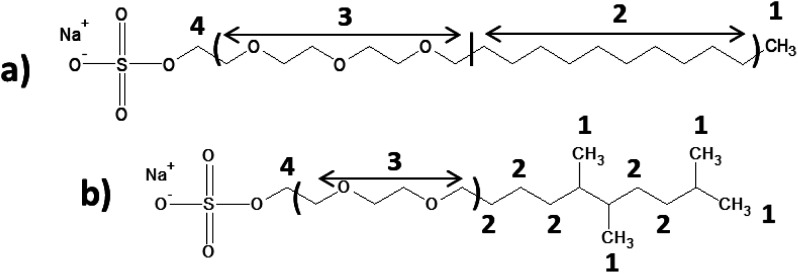					

### Perfume release from surfactant systems

**Fig. 3:**
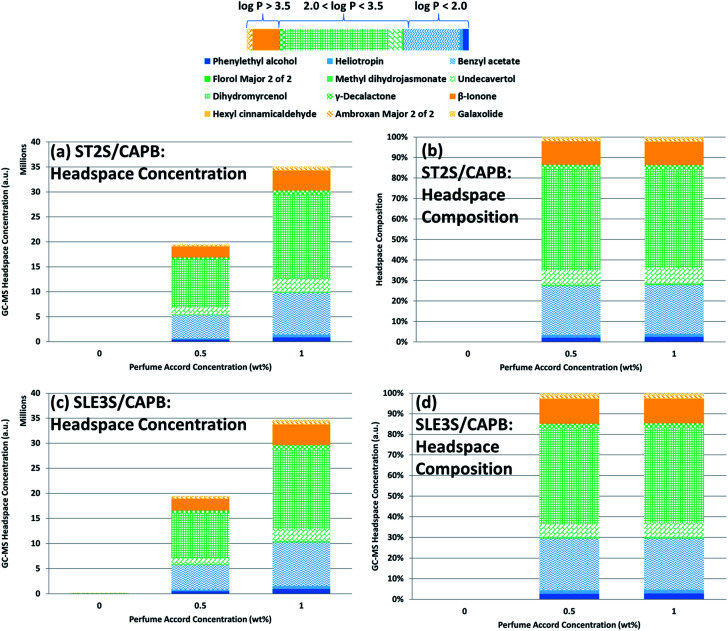
GC-MS headspace results as a function of perfume concentration: headspace concentrations for each PRM in (a) ST2S/CAPB and (c) SLE3S/CAPB systems; headspace composition of (b) ST2S/CAPB and (d) SLE3S/CAPB systems. The lipophilicity or hydrophilicity of each PRM is indicated by the color bar, with blue indicating that the PRM is hydrophilic, orange means the PRM is lipophilic, and green means it is intermediate. Within each column, the PRMs are ordered from lowest log *P* value (blue) to highest log *P* value (orange). The legend is below the color bar. In (b), there is a negligible amount of perfume in the headspace of the SLE3S/CAPB sample containing 0 wt% perfume owing to carry-over from the previously measured sample, and the corresponding headspace composition was omitted from (d) to prevent confusion.

**Fig. 4:**
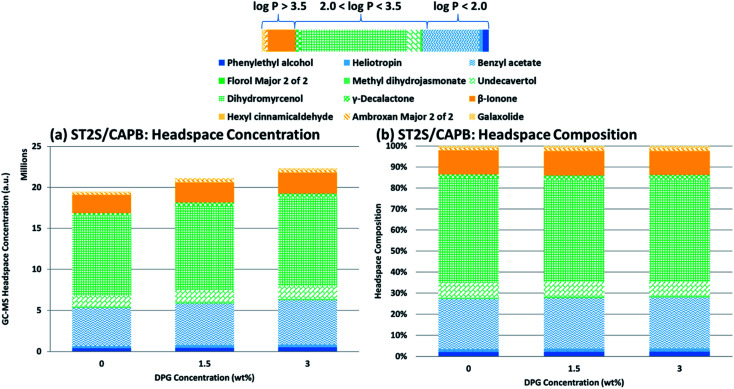
GC-MS headspace results as a function of DPG concentration: (a) headspace concentrations for each PRM in ST2S/CAPB systems; (b) corresponding headspace compositions. The lipophilicity or hydrophilicity of each PRM is indicated by the color bar, with blue indicating that the PRM is hydrophilic, orange means the PRM is lipophilic, and green means it is intermediate. The legend is below the color bar. In each column, the PRMs are ordered from lowest log *P* value (blue) to highest log *P* value (orange).

To study how effectively each PRM was released from each mixed-surfactant system as a function of perfume accord concentration and DPG concentration, the headspace concentrations were measured using GC-MS (Fig. 3 and 4). The PRM headspace concentrations increased less than two-fold when the perfume concentration was doubled in each mixed-surfactant system, *i.e.*, sample 2 *versus* sample 3 (Fig. 3a and b) and sample 7 *versus* sample 8 (Fig. 3c and d). Thus, the chemical activity of the PRMs increased as more perfume was added, resulting in the headspace concentrations increasing in accordance with Raoult's law. The headspace compositions did not change significantly with increasing perfume concentration for either surfactant system. The headspace compositions were dominated by the PRMs with intermediate log *P* values (2.0–3.5), followed by the highly hydrophilic PRMs and the highly lipophilic PRMs. The concentrations of the PRMs within each log *P* range varied greatly (*e.g.*, dihydromyrcenol had the highest headspace concentration among the PRMs with intermediate log *P* values), suggesting that the molecular structure of the PRM must be considered in addition to its log *P* value when attempting to improve the headspace concentration.

Fig. 4 shows the change in head space concentration and composition as the DPG concentration increased from 0 to 3 wt% (samples 2, 4, and 5). The total headspace concentration and the headspace concentrations of most PRMs increased upon DPG addition, despite the perfume concentration remaining constant. The headspace mainly consisted of PRMs with intermediate log *P* values (2.0–3.5), both in terms of the headspace concentration and the composition, followed by the highly hydrophilic PRMs and then the highly lipophilic PRMs. DPG addition caused the headspace concentration of each PRM to increase, but the relative ratio of the PRMs in the headspace remained constant, indicating that DPG addition did not preferentially improve the chemical activity of any particular PRM. The headspace concentration increasing without a significant change in its composition as DPG was added may have been caused by the micelles shrinking with DPG addition. Although the perfume is preferentially located in the center of the micelle based on the above NMR results, a smaller micelle would still have less volume in which the perfume could be solubilized. Therefore, as the micelles became smaller, the total amount of perfume the micelles could solubilize would also decrease; thus, the activities of every PRM would uniformly increase in kind, leading to the headspace concentration increasing without affecting the headspace composition.

The headspace concentrations of the PRMs within each log *P* range varied greatly (*e.g.*, dihydromyrcenol had the highest headspace concentration among the PRMs with intermediate log *P* values), as was the case when the perfume concentration was increased. Interestingly, the PRMs with the highest headspace concentrations in the intermediate and lipophilic log *P* groups (dihydromyrcenol and β-ionone, respectively) had the lowest molecular weights within these groups. For the hydrophilic PRMs, phenylethyl alcohol had the lowest molecular weight, but benzyl acetate had a significantly higher headspace concentration. The only structural difference between these PRMs is an acetate group *versus* a –CH_2_OH group. Within each log *P* group, the differences in the relative concentrations of each PRM in the perfume accord alone cannot explain the significant variations in the headspace concentrations. These observations confirm that the molecular structure of the PRM is also important to consider for improving perfume release from rinse-off systems.

## Conclusions

Past work on perfumes in surfactant systems has primarily focused on the effects of a single perfume molecule on the geometry of the surfactant self-assembly^15,16^ and where the perfume molecule localized within the assembly.^17,33^ In contrast, our previous work on a 3 PRM mixture investigated the effectiveness of the ST2S/CAPB mixed-surfactant system in creating single-phase microemulsions with perfume, the influence of perfume–perfume interactions on PRM localization within the self-assembly, and the effect of the perfume on the self-assembly.^7^

The present work is more industry relevant and built upon our previous work by employing an even more complex 12 PRM accord, consisting of PRMs with various structures and *c* log *P* values; by comparing two different mixed-surfactant systems with branched or linear tails; and by considering the cosolvent (DPG) concentration as a variable. SANS modeling showed that the geometry of the mixed-surfactant self-assemblies remained the same despite the tail structure, with both systems forming prolate ellipsoids. Perfume addition caused the micelle volumes in both systems to increase, but the ST2S/CAPB micelles were more strongly affected. Upon DPG addition, the ST2S/CAPB micelles became smaller and less elongated while retaining a prolate ellipsoidal geometry, which is consistent with findings of Jiang *et al.*^8^ and Padasala *et al.*^11^ The GC-MS headspace results showed that the headspace concentrations of all the PRMs increased as the perfume concentration increased, and also that the headspace concentrations increased slightly when DPG was added at a constant perfume concentration. The log *P* values and the PRM molecular structures appear to be important variables that influence the headspace concentration of each PRM. NMR analysis showed that the perfume accord preferentially localized amongst the surfactant tails in the branched-tail surfactant system, whereas localization along the tail and ethoxy groups was observed in the linear-tail surfactant system. Thus, perfume molecule localization can be influenced by more variables than those identified by Fan *et al.*^17^ and Fischer *et al.*^33^ Perfume–perfume interactions and the surfactant tail structure appear to be responsible for this behavior. These results show that the surfactant molecular structure and the changes to the system properties caused by cosolvent and perfume addition influence the geometry of the dispersed phase structures. Additionally, the release of active ingredients, such as fragrance, can be improved or hindered by the molecular structures and degree of lipophilicity/lipophobicity of the components as well as the physical properties of the overall system.

## Conflicts of interest

There are no conflicts to declare.

## Supplementary Material

RA-012-D2RA00688J-s001
