# Supplementary material for: Structural alterations of branched versus linear mixed-surfactant micellar systems with the addition of a complex perfume mixture and dipropylene glycol as cosolvent
Source: RSC Adv. 2022 May 17;12(24):14998–5007. doi: 10.1039/d2ra00688j (PMC9112669; doi:10.1039/d2ra00688j)
Supplement: RA-012-D2RA00688J-s001 [file RA-012-D2RA00688J-s001.pdf]

Electronic Supplementary Material (ESI) for RSC Advances.
This journal is © The Royal Society of Chemistry 2022

Supporting Information

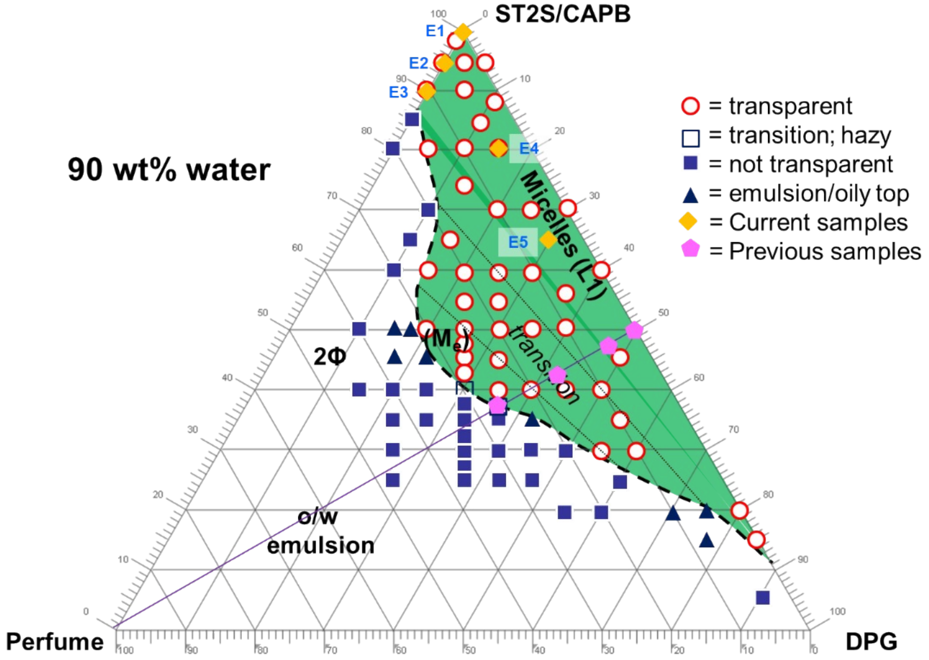

Figure S1: Approximate locations of samples E1-E5 (orange diamonds) on the pseudo-tertiary phase diagram at 90 wt% water, developed in our previous work. For comparison, the samples that were studied in the previous work are shown as pink pentagons. The single-phase region is colored green to aid in visualization. Each point on the diagram contains 90 wt% water, so the placement of each point on the diagram represents the composition of the remaining 10 wt%.

NMR Spectra

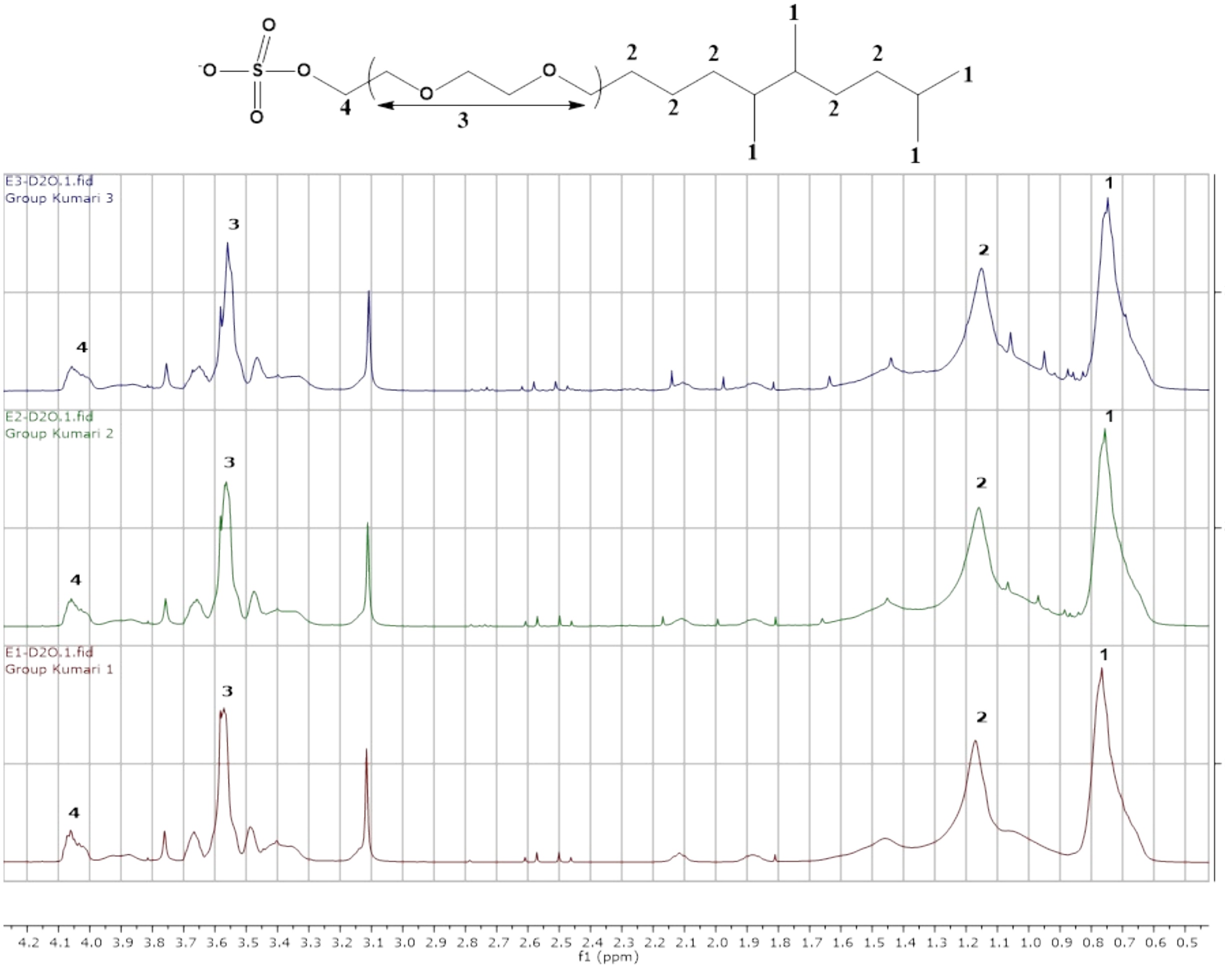

Figure S2: NMR spectra of the ST2S/CAPB system as a function of perfume concentration. Bottom: 0 wt% perfume; middle: 0.5 wt% perfume; top: 1 wt% perfume. Structure of the primary surfactant along with peak assignments are indicated above the spectra.

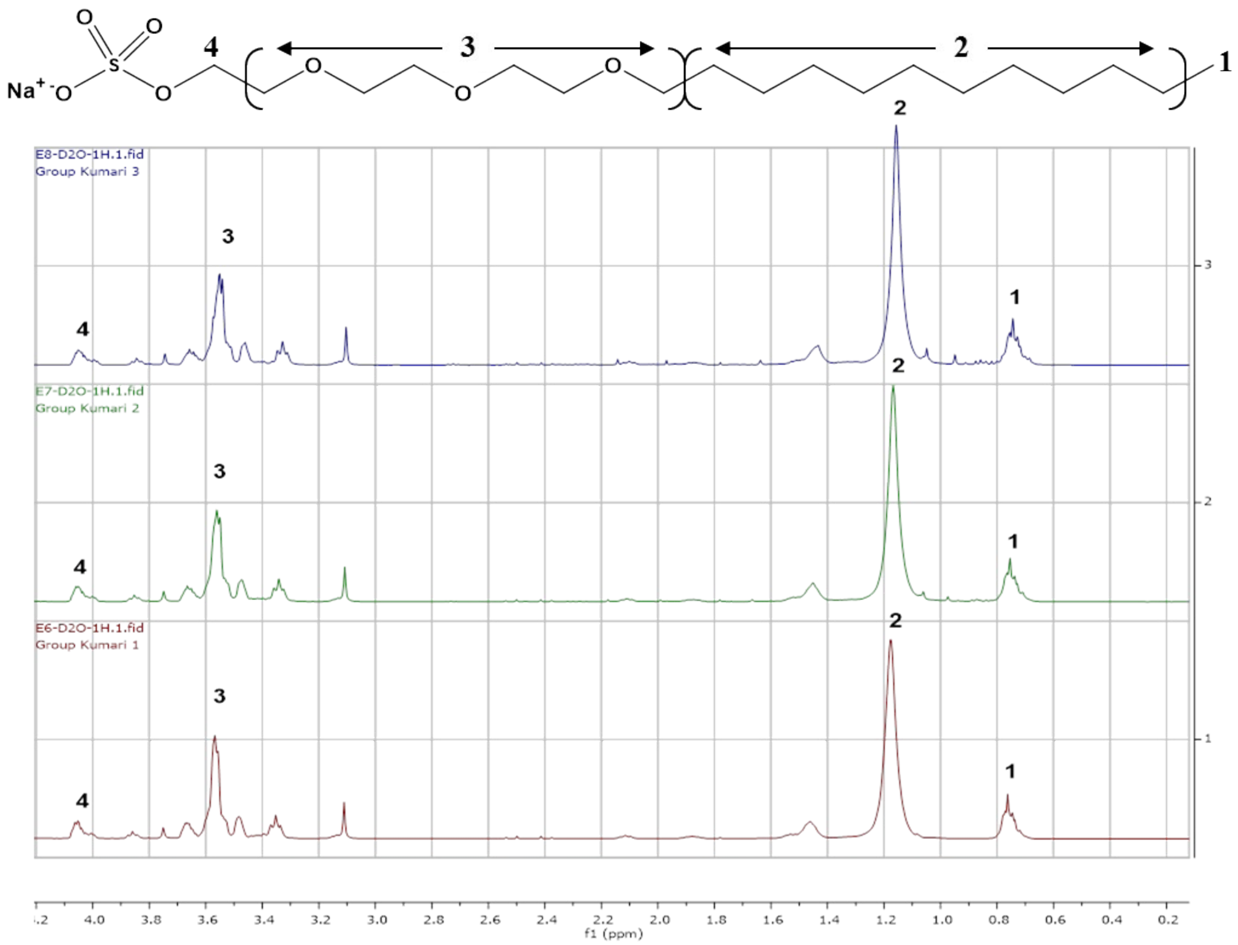

Figure S3: NMR spectra of the SLE3S/CAPB system as a function of perfume concentration. Bottom: 0 wt% perfume; middle: 0.5 wt% perfume; top: 1 wt% perfume. Structure of the primary surfactant along with peak assignments are indicated above the spectra.

SANS Fitting Results

A resolution-smeared version of the Uniform Ellipsoid form factor model coupled with the Hayter-Penfold MSA structure factor model was fitted to all 8 data sets in SasView 5.0 to investigate the micellar structure. Measurements were performed at 298 K. The scattering length densities (SLDs) for each component were calculated using the online neutron activation and SLD calculator provided by the NIST Center for Neutron Scattering and are shown in the tables below (https://www.ncnr.nist.gov/resources/activation/index.html?cutoff=0). The molecular formulae, densities, and SLDs for the raw materials, micellar phase, and solvent phase are shown in Table 1. Weighted averages of the materials assumed to be in the micelle or solvent portion of the structure were calculated and used in the SLD calculator to determine the micelle and solvent SLDs for samples E1-E8. The formulae, densities, and SLDs for the micellar phase and solvent phase are in Table 2. The formulae shown in Table 2 are in the format used in the SLD calculator. Sodium trideceth-2 sulfate (ST2S, for E1-E5), sodium laureth-3 sulfate (SLE3S, for E6-E8), cocamidopropyl betaine (CAPB), and the perfume accord were assumed to only be in the micellar phase. The components in the perfume accord were weight-averaged to obtain a weight-averaged density and molecular formula for the accord to be used in calculating the micelle SLD. Citric acid, dipropylene glycol (DPG), water, and deuterated water were assumed to only be in the solvent phase. The uncertainties shown for each fit is +/- one standard deviation from the result, and if the uncertainty equals 0 then the parameter was held constant at the specified value during the fitting procedure. In the model, $R_p > R_e$ denotes prolate ellipsoids while $R_e > R_p$ means the ellipsoids are oblate. The data sets were modeled with both cases of $R_p > R_e$ and $R_e > R_p$ to determine whether the micelles were oblate or prolate. Decisions were made based on which model had the most minimized residuals and how well the model fitted to the data visually, with special attention paid to the high-q region as it is most sensitive to the form factor. Both versions of the model fitted similarly well to samples E6-E8, so the final decision was based on the sqrt(χ^2/N) value.

Table S1: Molecular Formulae, densities, and SLDs for raw materials

Material	Molecular Formula	Density (g/cm^3)	SLD (Å^{-2})
ST2S	$C_{17}H_{35}NaO_6S$	1.1	3.75E-07
SLE3S	$C_{18}H_{37}NaO_7S$	1.15	4.67E-07
CAPB	$C_{19}H_{38}N_2O_3$	1.04	3.71E-07
Weight-averaged perfume accord	$C_{12.2}H_{19.6}O_{1.6}$	0.966	5.18E-07
Citric acid	$C_6H_8O_7$	1.54	2.45E-06
DPG	$C_6H_{14}O_3$	1.02	2.26E-07
Water	H_2O	1	-5.61E-07
Deuterated water	D_2O	1.11	6.38E-06

Table S2: Molecular Formulae, densities, and SLDs for micellar and solvent phases of samples E1-E8

Sample	Phase	Molecular Formula	Density (g/cm^3)	SLD ($Å^{-2}$)
E1	Micelle	85.36%wt $C_{17}H_{35}NaO_6S$ // $C_{19}H_{38}N_2O_3$	1.09	3.93e-7
	Solvent	92.30%wt D2O // 7.58%wt H2O // $C_6H_8O_7$	1.09	5.77e-6
E2	Micelle	80.02%wt $C_{17}H_{35}NaO_6S$ // 13.72%wt $C_{19}H_{38}N_2O_3$ // $C_{12.2}H_{19.6}O_{1.6}$	1.08	4.02e-7
	Solvent	92.80%wt D2O // 7.10%wt H2O // $C_6H_8O_7$	1.09	5.80e-6
E3	Micelle	75.32%wt $C_{17}H_{35}NaO_6S$ // 12.92%wt $C_{19}H_{38}N_2O_3$ // $C_{12.2}H_{19.6}O_{1.6}$	1.08	4.010e-7
	Solvent	93.30%wt D2O // 6.60%wt H2O // $C_6H_8O_7$	1.09	5.84e-6
E4	Micelle	80.02%wt $C_{17}H_{35}NaO_6S$ // 13.72%wt $C_{19}H_{38}N_2O_3$ // $C_{12.2}H_{19.6}O_{1.6}$	1.08	4.02e-7
	Solvent	90.10%wt D2O // 8.20%wt H2O // 1.6%wt $C_6H_{14}O_3$ // $C_6H_8O_7$	1.09	5.62e-6
E5	Micelle	80.02%wt $C_{17}H_{35}NaO_6S$ // 13.72%wt $C_{19}H_{38}N_2O_3$ // $C_{12.2}H_{19.6}O_{1.6}$	1.08	4.02e-7
	Solvent	90.10%wt D2O // 6.50%wt H2O // 3.3%wt $C_6H_{14}O_3$ // $C_6H_8O_7$	1.09	5.63e-6
E6	Micelle	90.00%wt $C_{18}H_{37}NaO_7S$ // $C_{19}H_{38}N_2O_3$	1.14	4.58e-7
	Solvent	70.00%wt D2O // 29.90%wt H2O // $C_6H_8O_7$	1.07	4.14e-6
E7	Micelle	84.37%wt $C_{18}H_{37}NaO_7S$ // 9.40%wt $C_{19}H_{38}N_2O_3$ // $C_{12.2}H_{19.6}O_{1.6}$	1.13	4.63e-7
	Solvent	70.30%wt D2O // 29.50%wt H2O // $C_6H_8O_7$	1.07	4.16e-6
E8	Micelle	79.41%wt $C_{18}H_{37}NaO_7S$ // 8.80%wt $C_{19}H_{38}N_2O_3$ // $C_{12.2}H_{19.6}O_{1.6}$	1.12	4.66e-7
	Solvent	70.70%wt D2O // 29.10%wt H2O // $C_6H_8O_7$	1.07	4.19e-6

E1 (no perfume or DPG, ST2S/CAPB System)

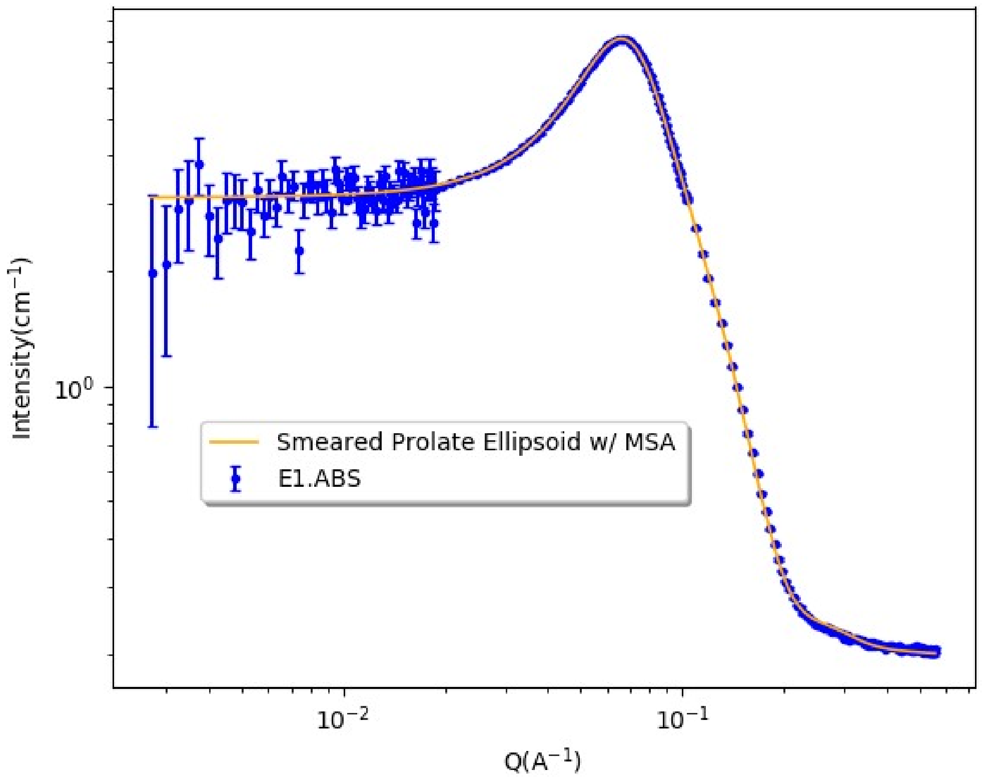

Scale	1
Volume fraction	0.085 ± 8.61e-5
R_p (rotation axis) (Å)	54.82 ± 0.14
R_e (Å)	18.21 ± 0.01
Polydispersity R_e	0.15 ± 3.79e-4
SLD ellipsoid (Å^{-2})	3.93e-07
SLD solvent (Å^{-2})	5.77e-06
Charge	14.62 ± 0.05
Monovalent salt (M)	0
Temperature (K)	298
Dielectric constant	78.0
Incoherent bkg. (cm^{-1})	0.198 ± 2.30e-4
Sqrt(X^2/N)	1.77

E2 (0.5 wt% perfume, no DPG, ST2S/CAPB System)

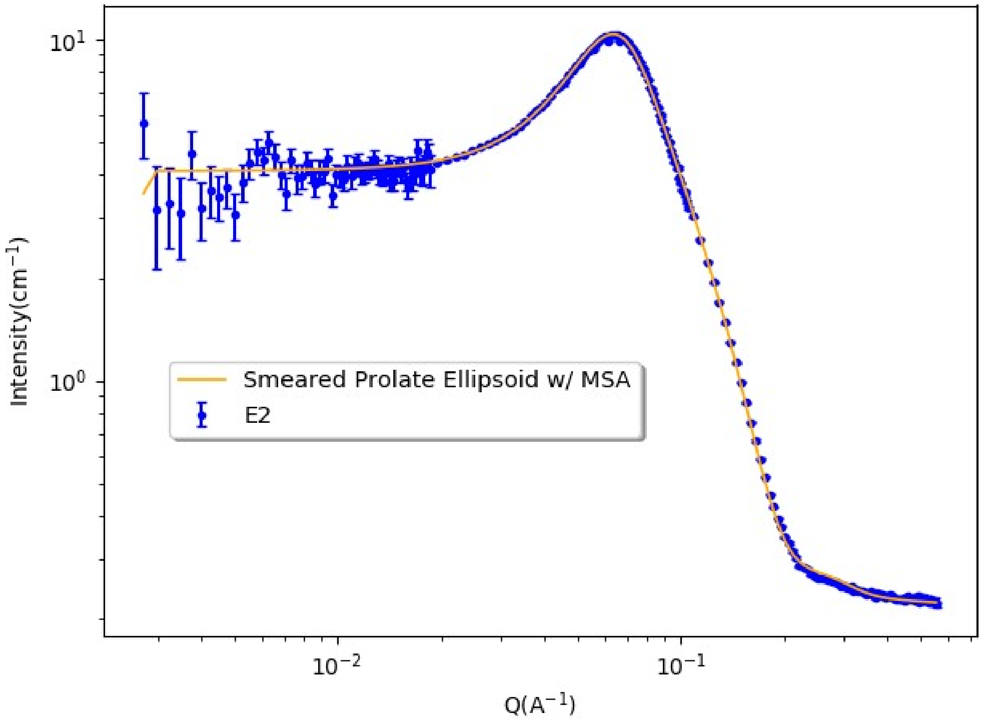

Scale	1
Volume fraction	0.10 ± 9.10e-5
R_p (rotation axis) (Å)	66.86 ± 0.17
R_e (Å)	18.70 ± 0.01
Polydispersity R_e	0.14 ± 3.32e-4
SLD ellipsoid (Å^{-2})	4.02e-07
SLD solvent (Å^{-2})	5.80e-06
Charge	15.80 ± 0.06
Monovalent salt (M)	0
Temperature (K)	298
Dielectric constant	78.0
Incoherent bkg. (cm^{-1})	0.22 ± 2.58e-4
Sqrt(X^2/N)	2.90

E3 (1 wt% perfume, no DPG, ST2S/CAPB System)

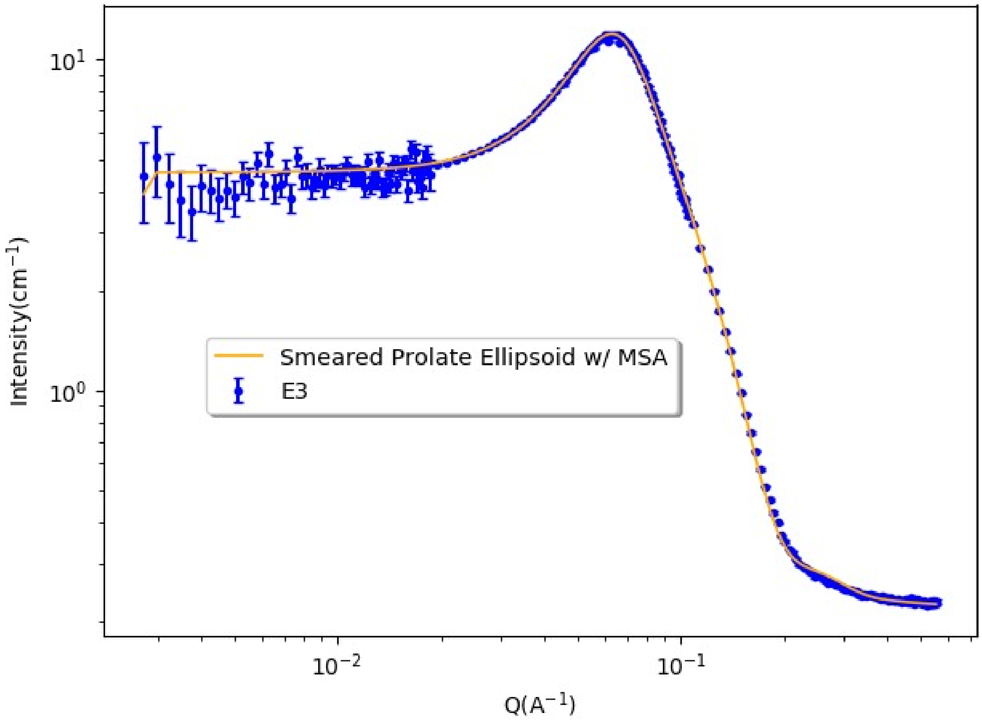

Scale	1
Volume fraction	0.11 ± 9.36e-5
R_p (rotation axis) (Å)	68.20 ± 0.17
R_e (Å)	19.43 ± 0.01
Polydispersity R_e	0.13 ± 3.09e-4
SLD ellipsoid (Å^{-2})	4.10e-07
SLD solvent (Å^{-2})	5.84e-06
Charge	16.76 ± 0.06
Monovalent salt (M)	0
Temperature (K)	298
Dielectric constant	78.0
Incoherent bkg. (cm^{-1})	0.22 ± 2.64e-4
Sqrt(X^2/N)	3.52

E4 (0.5 wt% perfume, 1.5 wt% DPG, ST2S/CAPB System)

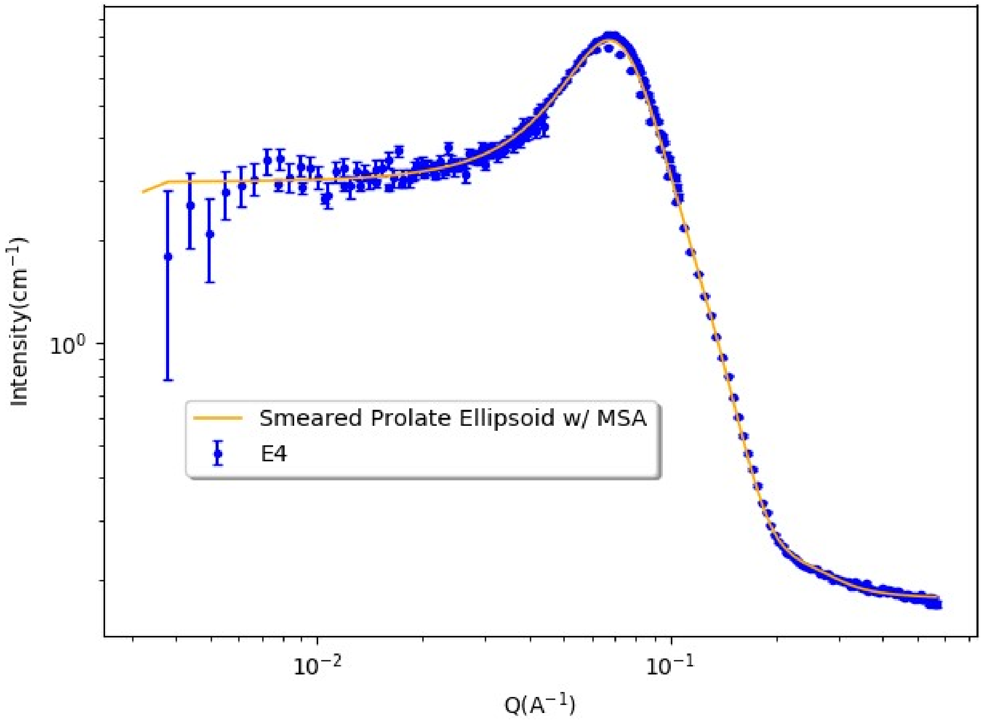

Scale	1
Volume fraction	0.080 ± 9.43e-5
R_p (rotation axis) (Å)	45.74 ± 0.14
R_e (Å)	19.25 ± 0.02
Polydispersity R_e	0.17 ± 8.20e-4
SLD ellipsoid (Å^{-2})	4.02e-07
SLD solvent (Å^{-2})	5.62e-06
Charge	15.08 ± 0.10
Monovalent salt (M)	0
Temperature (K)	298
Dielectric constant	77.1
Incoherent bkg. (cm^{-1})	0.18 ± 2.36e-4
Sqrt(X^2/N)	3.64

E5 (0.5 wt% perfume, 3 wt% DPG, ST2S/CAPB System)

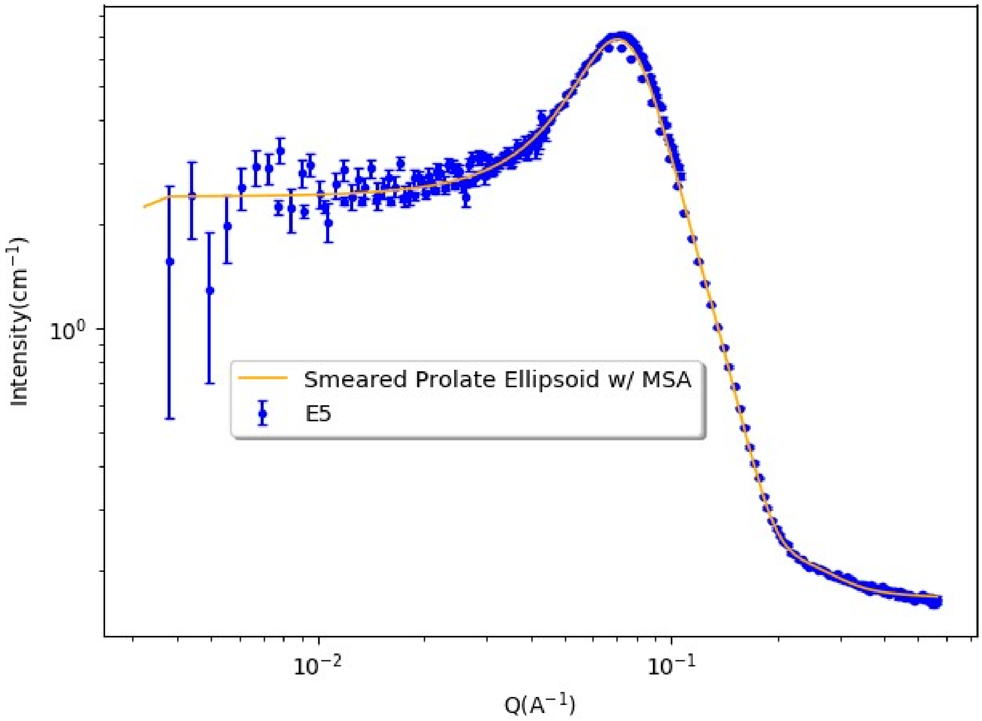

Scale	1
Volume fraction	0.075 ± 9.04e-5
R_p (rotation axis) (Å)	40.88 ± 0.12
R_e (Å)	18.89 ± 0.02
Polydispersity R_e	0.16 ± 8.34e-4
SLD ellipsoid (Å^{-2})	4.02e-07
SLD solvent (Å^{-2})	5.63e-06
Charge	15.98 ± 0.10
Monovalent salt (M)	0
Temperature (K)	298
Dielectric constant	76.1
Incoherent bkg. (cm^{-1})	0.17 ± 2.29e-4
Sqrt(X^2/N)	3.50

E6 (no perfume or DPG, SLE3S/CAPB System)

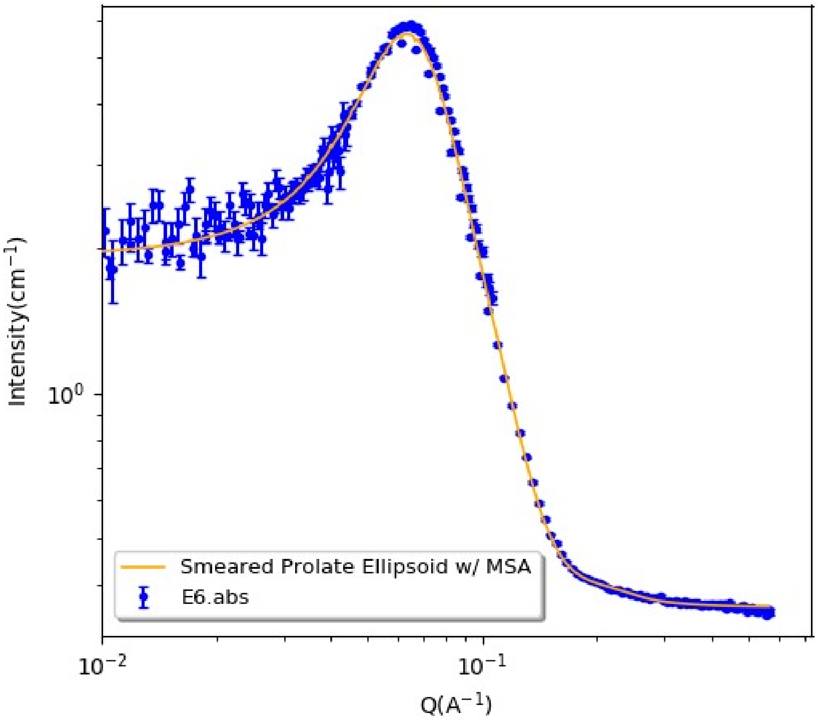

Scale	1
Volume fraction	0.079 ± 1.28e-4
R_p (rotation axis) (Å)	41.08 ± 0.16
R_e (Å)	22.91 ± 0.04
Polydispersity R_e	0.16 ± 1.35e-3
SLD ellipsoid (Å^{-2})	4.58e-07
SLD solvent (Å^{-2})	4.14e-06
Charge	16.65 ± 0.12
Monovalent salt (M)	0
Temperature (K)	298
Dielectric constant	78.5
Incoherent bkg. (cm^{-1})	0.36 ± 2.94e-4
Sqrt(X^2/N)	3.04

E7 (0.5 wt% perfume, no DPG, SLE3S/CAPB System)

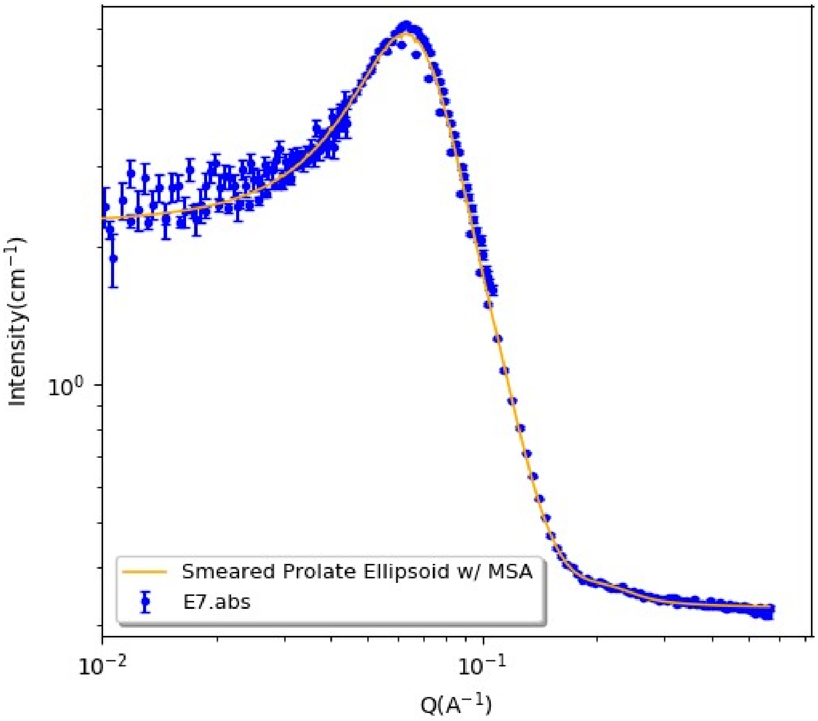

Scale	1
Volume fraction	0.081 ± 1.49e-4
R_p (rotation axis) (Å)	42.95 ± 0.20
R_e (Å)	23.23 ± 0.04
Polydispersity R_e	0.14 ± 3.84e-3
SLD ellipsoid (Å^{-2})	4.63e-07
SLD solvent (Å^{-2})	4.16e-06
Charge	20.15 ± 0.78
Monovalent salt (M)	0.011 ± 1.64e-3
Temperature (K)	298
Dielectric constant	78.5
Incoherent bkg. (cm^{-1})	0.33 ± 2.85e-4
Sqrt(X^2/N)	3.14

E8 (1 wt% perfume, no DPG, SLE3S/CAPB System)

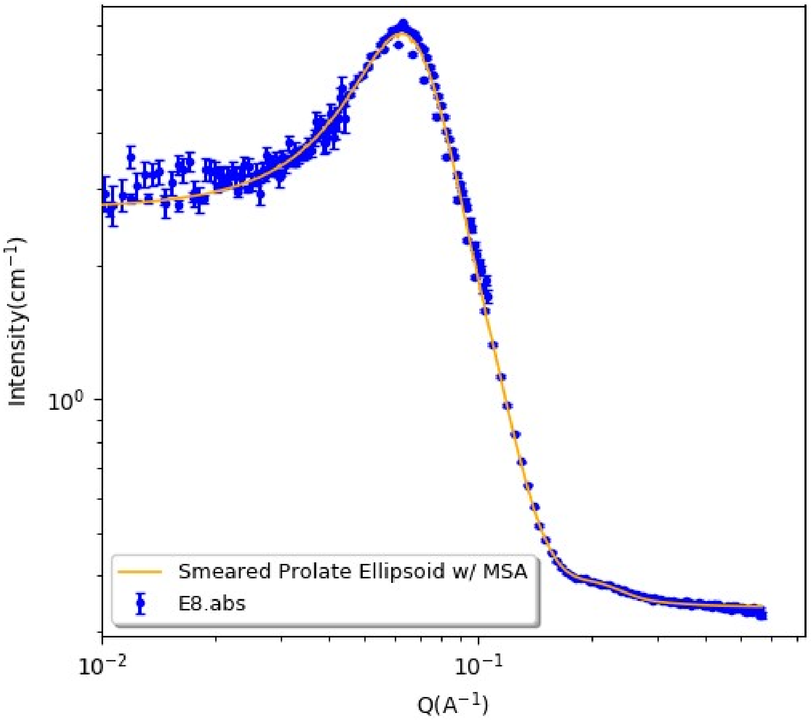

Scale	1
Volume fraction	0.088 ± 1.57e-4
R_p (rotation axis) (Å)	44.68 ± 0.20
R_e (Å)	23.99 ± 0.04
Polydispersity R_e	0.13 ± 3.53e-3
SLD ellipsoid (Å^{-2})	4.66e-07
SLD solvent (Å^{-2})	4.19e-06
Charge	23.32 ± 1.03
Monovalent salt (M)	0.019 ± 2.20e-3
Temperature (K)	298
Dielectric constant	78.5
Incoherent bkg. (cm^{-1})	0.34 ± 2.91e-4
Sqrt(X^2/N)	3.10
